# Clinical effect of aerobic exercise training in chronic obstructive pulmonary disease: A retrospective study

**DOI:** 10.1097/MD.0000000000035573

**Published:** 2023-10-20

**Authors:** Qigang Zeng, Wangwang Liao, Wentao Fang, Shuling Liu, Chenxia Duan, Yong Dai, Chenggong Wei

**Affiliations:** a Guangdong Hospital of Integrated Traditional Chinese and Western Medicine, Guangdong Province, China; b Guangzhou University of Chinese Medicine, Guangdong Province, China; c Affiliated Guangdong Hospital of Integrated Traditional Chinese and Western Medicine of Guangzhou University of Chinese Medicine, Guangdong Province, China.

**Keywords:** aerobic exercise training, chronic obstructive pulmonary disease, clinical retrospective trial, Yi Jin Jing

## Abstract

Aerobic exercise training is a kind of pulmonary rehabilitation for lung diseases. This was a retrospective study to assess the efficacy of aerobic exercise training in chronic obstructive pulmonary disease (COPD) at a stable stage. A total of one hundred and fifty-six stable COPD patients who had accepted self-education only or self-education combined with an aerobic exercise training between January 2017 to January 2019 were reviewed retrospectively. A total of 79 patients who had received self-education combined with an aerobic exercise training schedule comprised the aerobic exercise training group (AET group) and 77 patients who had received self-education only were regarded as the education group (EDU group). The acute incidence rate in AET group was 7.6% better than that in EDU group 20.7% (*P* < .05). The AET group patients expressed higher levels of 6 minutes walking distance (6MWD) (*P* < .05) and better evaluations of both lung function (*P* < .05) and T lymphocyte immune response (*P* < .05), as well as significantly decreased chronic obstructive pulmonary disease assessment test (CAT) scores and modified British medical research council (mMRC) grades (*P* < .05). Patients in EDU group did not report any changes in any of these characteristics. The aerobic exercise training intervention contributed to an increasing in 6MWD and decrease in CAT scores and mMRC grades, as well as improving the T lymphocyte immune response in stable COPD patients.

## 1. Introduction

Chronic obstructive pulmonary disease (COPD) is becoming one of the most important health problems worldwide, leading to high social costs and economic burdens.^[1]^ COPD is defined as a common, preventable and curable disease by the Global Initiative for Chronic Obstructive Pulmonary Disease (GOLD; updated 2022) and is mainly characterized by reversible airflow limitation.^[2]^ COPD is defined as a chronic inflammatory disorder of the airways in which chronic inflammatory cells play an important role.^[3]^ The 2022 version of GOLD advises COPD treatments based on the severity and clinical symptoms, as well as the risk of acute exacerbation (AE), instead of relying heavily on airflow limitation assessment. Therefore, COPD remedies should concentrate more on avoiding risk elements, alleviating symptoms, and guarding against AE recurrence. However, stable COPD patients, among the current treatments, mainly focus on pharmacotherapy, including the inhalation or administration of corticosteroids, inhalation of short or long acting β2 agonists and other pharmacotherapy. Medication are known to have an effect on relieving symptoms and improving physical functioning. Patients with stable COPD, still feel fatigued, dizzy and often shortness of breath despite all medical treatments.^[4]^ In addition, all the treatments mentioned above may lead to certain side effects of drugs, such as heart toxicity, risks of pneumonia, or gastrointestinal adverse reactions.^[5]^ The pathophysiological basis of fatigue in COPD is complex, and its precise mechanisms remain unknown.^[2]^

Thus far, there is a pressing need to establish certain strategies instead of medical treatment, which could have a certain effect in improving clinical symptoms, lower lung function decline, reduced mortality, and shortened hospitalization time. It has long been postulated that functional inspiratory muscle weakness is a contributor to dyspnea in advanced COPD.^[6]^ In addition, it was assumed that the central system could generate a given force or tension when the weakened muscles existed.^[7]^

Aerobic exercise has been reported to successfully improve cardio respiratory fitness, resulting in a significant increase in physical activity levels.^[8]^ In fact, involvement in aerobic exercise may be beneficial to patients with heart failure, pulmonary diseases, and unexplained physical symptoms.^[9]^ In stable COPD patients, there are certain evidences showed remarkable improvements in cardio respiratory fitness and the clinical effectiveness have been reported after exercise training during the AE.^[10,11]^ However, few studies have suggested that rehabilitative exercises are required by patients at a stable stage.

This retrospective study aimed to evaluate the clinical effectiveness of aerobic exercise training in altering the severity of respiratory fatigue and improving physical activity. It was hypothesized that participation in aerobic exercise training could help stable COPD patients ameliorate the body condition and improve their symptoms.

## 2. Methods

### 2.1. Participant inclusion

Patients who had accepted self-education only or self-education combined with an aerobic exercise training between January 2017 to January 2019 were reviewed retrospectively.

The diagnostic criteria for stable COPD were as follows: patients with stable symptoms such as cough, sputum production, or dyspnea for 4 to 6 weeks with no AE; pulmonary function test of forced expiratory volume in 1 second/forced vital capacity (FEV1/FVC) ≤ 70% after bronchodilators use.

Inclusion criteria: had accepted conventional treatments (as exhibited in Table 1); had accepted self-education only, or self-education combined with aerobic exercise-training.

**Table 1:** Conventional treatment for chronic obstructive pulmonary disease according to the recommendations in chronic obstructive pulmonary disease.

Strategy	First choice	Substitution	Others
A	SAMA prn or SABA prn	LAMA or LABA or SABA + SAMA	Theophylline
B	LAMA or LABA	LAMA or LABA	Theophylline SABA +/or SAMA
C	ICS + LABA or LAMA	LABA or LAMA	Theophylline SABA +/or SAMA Consider PDE4-I
D	ICS + LABA or LAMA	ICS + LABA + PDE4-I LAMA + PDE4-I ICS + LAMA LAMA + LABA	Theophylline SABA +/or SAMA Carbocisteine

ICS = inhaled glucocorticoid, LABA = long acting β2-receptor agonist, LAMA = long acting anticholinergic drug, PDE4-I = phosphodiesterase-4 inhibitor, SABA = short acting β 2-receptor agonist, SAMA = short acting anticholinergic drug; prn, as needed.

Exclusion Criteria: failure to follow-up visit; a lack of basic information; incomplete clinical data.

### 2.2. Study design

Patients who had received self-education only were regarded as the education group (EDU group). Patients who had accepted self-education combined with an aerobic exercise training schedule comprised the aerobic exercise training group (AET group).

AET group: Receiving conventional therapy, which completely met the guideline of the GOLD (Table 1), and self-education.

EDU group: Combination the therapies of AET group and aerobic exercise training.

The following data were collected: Basic information: age, course of COPD, gender, smoking history, and higher education, with the evaluation of baseline. The occurrence or absence of AECOPD. Pulmonary function; 6 minutes walking distance (6MWD), chronic obstructive pulmonary disease assessment test (CAT) score, modified British medical research council (mMRC) dyspnea grade, T lymphocyte subsets cluster of differentiation 4 (CD4+), cluster of differentiation 8 (CD8+), cluster of differentiation 4 ratio cluster of differentiation 8 (CD4+/CD8+) in blood. The data of this retrospective study was approved by the Institutional Research Ethics Committee of the Guangdong Hospital of Integrated Traditional Chinese and Western Medicine. The data were anonymized, and the requirement for informed consent was waived.

Self-education: Including lectures on chronic obstructive pulmonary disease development, drug use, long-term home-based oxygen therapy, social resources, advance directives, and direction of atomizing inhalation device, totally 16 education lessons, 20 minutes per session, twice a week over 8 weeks.

Aerobic exercise training: The program containing treadmill and Yi Jin Jing. The treadmill exercise was completed in hospital training room with medically surveillance. In particularly, the Yi Jin Jing exercise was a home-base rehabilitation course. The target exercise intensity control was investigated by the cardiopulmonary exercise experiment at the beginning of the exercise training session, and it ranged from about 70% to 80% of each patient heart rate (HR) reserve during aerobic classes. Target heart rate range was calculated as: [(peak HR-resting HR)*(%intensity)]+(resting HR), peak HR = (220-age), according to the means of Karvonen.^[12]^ During aerobic classes, dyspnea and fatigue, oxyhemoglobin saturation, and heart rate were continuously monitored. With all the above data, we could analyze these data comprehensively and systematically, set up the database, and keep each patient target exercise intensity as close as possible to their own target heart range.

Treadmill: During training, patients first warmed up for 5 minutes at a power rate of 10W, and it was advisable to increase the heart rate by approximately 20 times/min. With an increase in the exercise load, it would reach 15W. The maximum heart rate was suitable for patients with a 50% to 70% target rate, and the time to reach the target rate was maintained for 10 minutes. In the last 5 minutes, the exercise load was reduced to 10W, and the rest of the training was resumed. Each training session lasted approximately 40 minutes, twice a week for 8 weeks.

Yi Jin Jing: Yi Jin Jing, a method of nurturing the health of traditional Chinese medicine, is widely used to strengthen the body, mediate the yin and yang of the viscera and improve immune capability by diaphragm-contraction deep-breathing exercises that can improve vital capacity. The versions of Yi Jin Jing in this study, a home-based prescribed pulmonary rehabilitation, mainly include 12 types: Wei Tuo Presenting the Pestle 1; Wei Tuo Presenting the Pestle 2; Wei Tuo Presenting the Pestle 3; Plucking a Star and Exchanging a Star Cluster; Pulling Nine Cows by their Tails; Displaying Paw-Style Palms like a White Crane Spreading Its Wings; Nine Ghosts Drawing Swords; Three Plates Falling on the Floor; Black Dragon Displaying Its Claws; Tiger Springing on Its Prey; Bowing Downin Salutation, and Swinging the Tail. For more details, please see the *Chinese Health Qigong-Yi Jin Jing* (complied by the Chinese Health Qigong Association, FOREIGN LANGUAGES PRESS, 2007-01, ISBN:9787119047782). The patients performed every 2 days in a week, once a day, about 30 minutes each time, which was supervised by online videos or phone calls.

### 2.3. Assessment

Number of AEs: To record the quantity of the patients with the exacerbation of cough, sputum production, or dyspnea.

Lung function: Differences in flow and volume responses including FEV1 and FVC after the inhalation of a bronchodilator (100 μg salbutamol sulfate inhalation aerosol; Glaxo Wellcome, S.A., Aranda de Duero Burgos, Spain). The data were measured using a COSMED spirometer (Quark PFT4, Inc., Longmont, CO, USA).

6MWD score: The 6MWD test was assessed based on a method used in an antecedent study.^[13]^ Certain parameters such as heart rate, blood pressure, and oxygen saturation were measured before and after the test. The Borg Scale score was also calculated.

CAT score: It includes 8 questions: cough, sputum, chest tightness, activity ability, daily living ability, going out ability, sleep and energy. The score of each question is 0 to 5, with a total score of 0 to 40. The higher the score, the worse the health status of patients.

mMRC dyspnea score: All patients had dyspnea scores according to the mMRC dyspnea score grade.^[14]^ The mMRC dyspnea score provides a certain grade system ranges from 1 to 5 grades, which was scaled according to various physical activities that may cause different grades of dyspnea: grade 0, no remarkable dyspnea, unless during aggravating activities; grade 1, shortness of breathing on fast walking or on short range walking distance; grade 2, slower walking than peers owning to breathing problems or needing to stop to rest when walking on flat ground at normal speed; grade 3, need to stop because of dyspnea within 100-m walk or after a few minutes of walking on flat ground; and grade 4, unable to finish even light activities or leave the house because of significant dyspnea, or feel shortness of breath at most of the time.^[15]^

T lymphocyte subsets: The T lymphocyte subsets CD4+, CD8+, and CD4+/CD8+, in the blood measured by flow cytometry (FACS Calibur, Becton, Dickinson and Company, USA). All the participants took 2 mL of venous blood plus 0.4 mL EDTA anticoagulation in the morning prior to and at the end of the trial. Took 100 μL of the blood specimen, using monoclonal antibodies labeled with the simule Test reagent, which contained 2-color fluorescein. After training for 20 minutes in 4°C, avoid light environment, 2 mL RBC pyrolysis liquid was added and blending and cultivate 10 minutes at room temperature. Finally, the sample was washed twice with PBS, and 200 mL of PBS was mixed and immediately check it.^[16]^

### 2.4. Statistical analysis

All data were presented as the mean ± standard deviation and were calculated and analyzed using the statistical method of 2 independent samples *t* test using SPSS (version 26.0). Differences in the severity of the first AECOPD episode were calculated using Fisher exact test. Data such as gender, duration of smoking history and educational background were statistically calculated using the χ^2^ test for the contingency table. mMRC and CAT scores were analyzed using Welch test. Statistical significance was set at *P* < .05.

## 3. Results

### 3.1. Demographic and clinical characteristics

**Figure 1.**
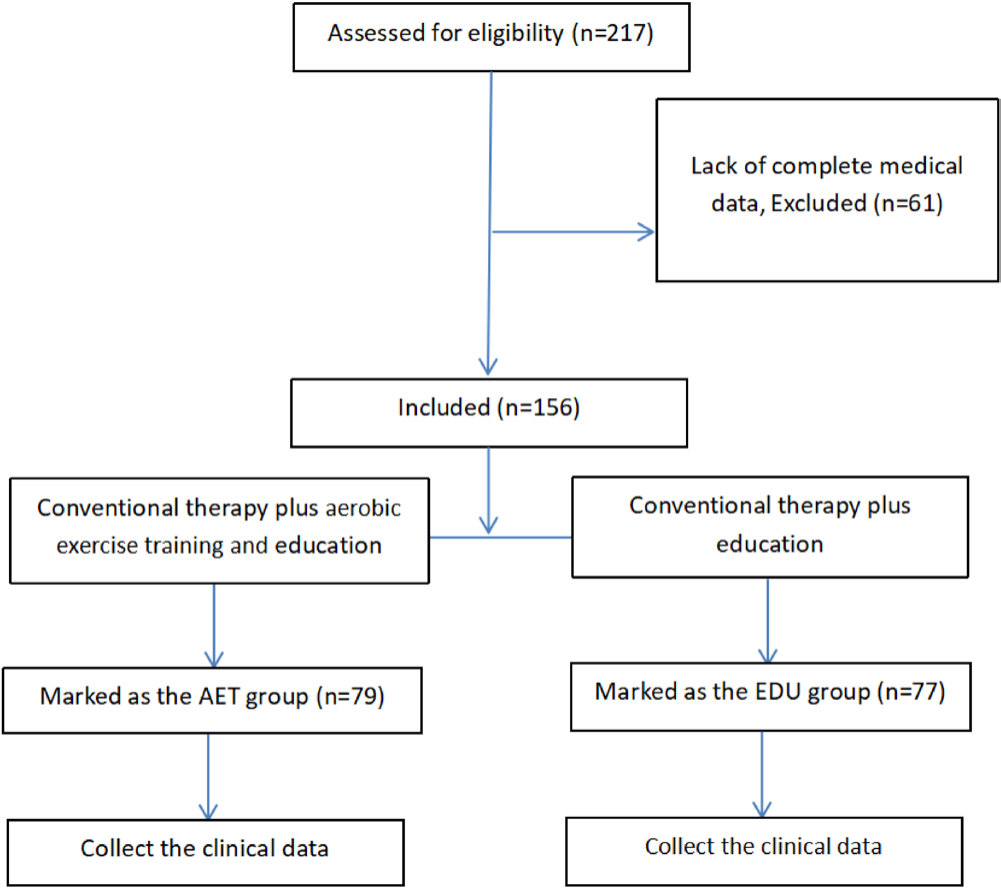
Flow chart displaying the participation throughout the study process.

A total of 217 patients met the retrospective analysis (Fig. 1), 61 of whom were excluded due to a lack of complete medical data. Seventy-nine patients treated with conventional medicine, self-education and aerobic exercise training were marked as the AET group, and 77 patients treated with conventional medicine and self-education were marked as the EDU group. The basic data of all patients are presented in Table 2. No significant differences were observed in either group of patients.

**Table 2:** Baseline characteristics of the enrolled patients.

Factors	AET (n = 79)	EDU (n = 77)
Age, yr	62.75 ± 5.62	63.13 ± 5.21
Course of COPD, yr	7.06 ± 2.35	6.44 ± 2.78
Gender, n (%)^a^		
Male	69 (87.3%)	68 (88.3%)
Female	10 (12.7%)	9 (11.7%)
Smoking history, n (%)^a^		
Yes	67 (84.8%)	66 (85.7%)
No	12 (15.2%)	11 (14.3%)
Higher education^a^		
Yes, n (%)	16 (20.3%)	18 (23.4%)
No, n (%)	63 (79.7%)	59 (76.6%)
Lung function		
FEV1 (L)	1.01 ± 0.30	1.10 ± 0.50
FVC (L)	2.24 ± 0.31	2.25 ± 0.63
CAT	16.69 ± 3.94	16.44 ± 3.39
6MWD (m)	541.19 ± 79.28	535.69 ± 65.89
mMRC dyspnea score	1.94 ± 0.68	1.81 ± 0.66

Data are presented as mean ± standard deviation and analyzed with 2 independent sample *t* tests. The data between AET group versus EDU group have no statistical difference (*P* > .05).

6MWD = 6 minutes walking distance, CAT = chronic obstructive pulmonary disease assessment test, COPD = chronic obstructive pulmonary disease, FEV1 = forced expiratory volume in 1 second, FVC = forced vital capacity, mMRC = modified British medical research council.

a Data were analyzed using the χ^2^ test for contingency table.

### 3.2. Occurrence of AECOPD

A comparison of AE occurrence between the 2 groups showed a significant difference. Six patients in the AET group and 16 patients in the EDU group reported AE occurrence, with a higher AE occurrence rate in the EDU group (*P* < .05). Accordingly, the results suggested that aerobic exercise training courses could significantly diminished the exacerbation of COPD and decrease the duration of the AE period (Table 3).

**Table 3:** Primary characteristics of AECOPD.

Index	AET (n = 79)	EDU (n = 77)	χ^2^	*P* value
Exacerbation cases, n (%)^a^	6 (7.6%)	16 (20.8%)*	5.595	.018

AECOPD = acute exacerbations of chronic obstructive pulmonary disease.

a Data were analyzed with χ^2^ test for contingency table.

* *P* < .05 for difference in change score between AET group versus EDU group.

### 3.3. Pulmonary function, 6 MWD, CAT scores, and mMRC grade

**Figure 2.**
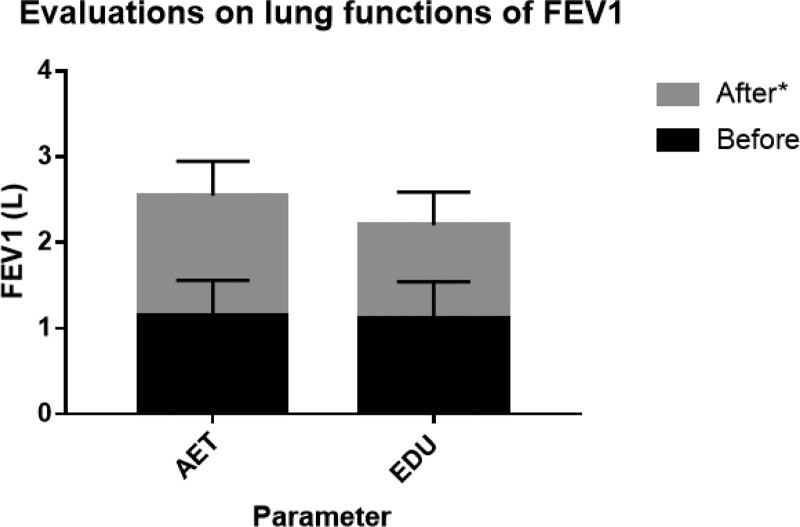
Evaluation on lung functions of FEV1. Change in lung functions of FEV1 in response to 6-weeks of aerobic exercise training plus education (AET) or education alone (EDU). *P < .05 for difference in change score between AET group versus EDU group. AET group = aerobic exercise training plus self-education group, EDU group = self-education only group, FEV1 = forced expiratory volume in 1 s.

**Figure 3.**
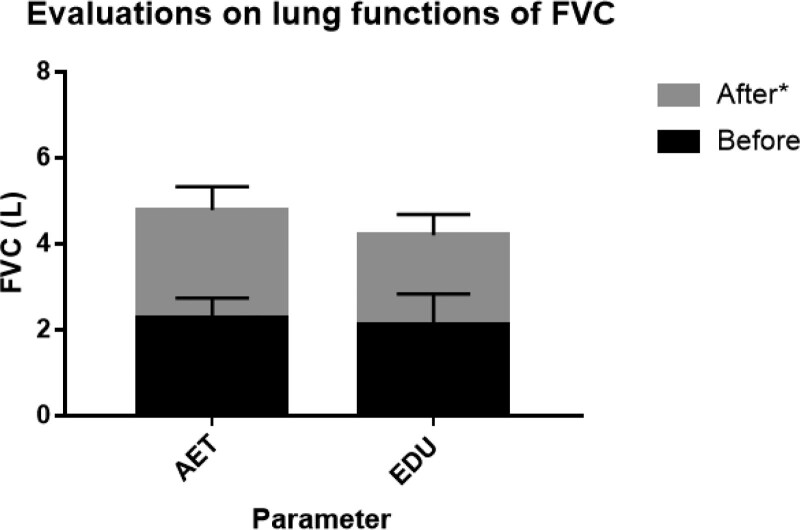
Evaluation on lung functions of FVC. Change in lung functions of FVC in response to 6-weeks of aerobic exercise training plus education (AET) or education alone (EDU). *P < .05 for difference in change score between AET group versus EDU group. AET group = aerobic exercise training plus self-education group, EDU group = self-education only group, FVC = forced vital capacity.

Pulmonary function was assessed before and at the end of the aerobic exercise training. The values of FEV1 and FVC in the AET group increased significantly when compared with those in the EDU group and reached the best situation after aerobic exercise training (Table 4; Figs. 2 and 3). A comparison of the difference in pulmonary function before and after the aerobic exercise training, a more obvious amelioration in the AET group.

**Table 4:** Evaluations on lung functions.

Parameter	Time	EDU (n = 77)	AET (n = 79)	T-value	*P* value
FEV1	After intervention	1.11 ± 0.45	1.42 ± 0.34*	5.28	<.01
FVC	After intervention	2.32 ± 0.52	2.66 ± 0.38*	4.71	<.01

Data were analyzed with 2 independent samples *t* test and the mean values are presented. FEV1, forced expiratory volume in 1 second; FVC, forced vital capacity.

FEV1 = forced expiratory volume in 1 second, FVC = forced vital capacity.

* *P* < .05 for difference in change score between AET group versus EDU group.

**Figure 4.**
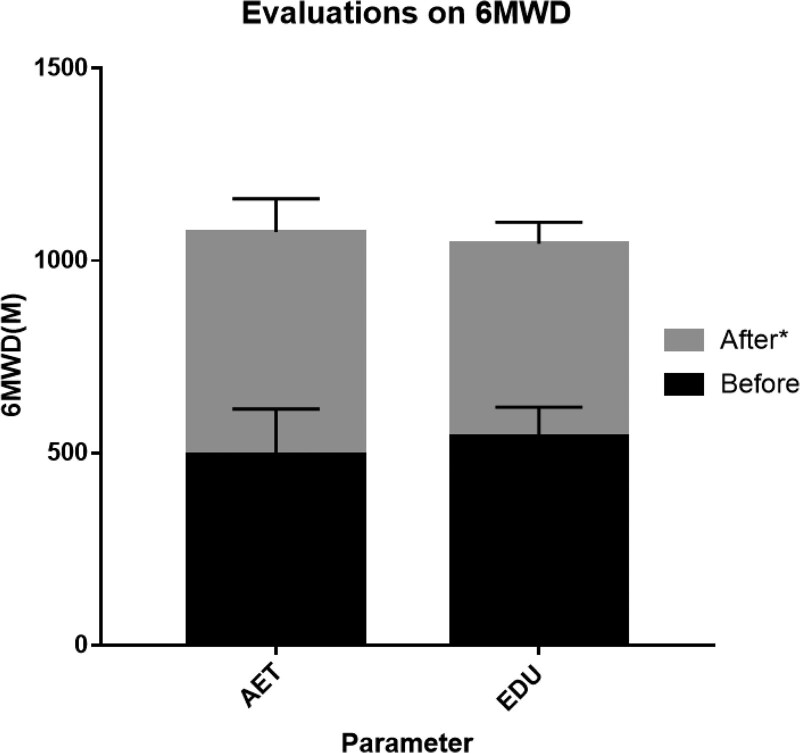
Evaluation on lung functions of 6MWD. Change in 6 minutes walking distance (6MWD) in response to 6-weeks of aerobic exercise training plus education (AET) or education alone (EDU). *P < .05 for difference in change score between AET group versus EDU group. AET group = aerobic exercise training plus self-education group, EDU group = self-education only group.

**Figure 5.**
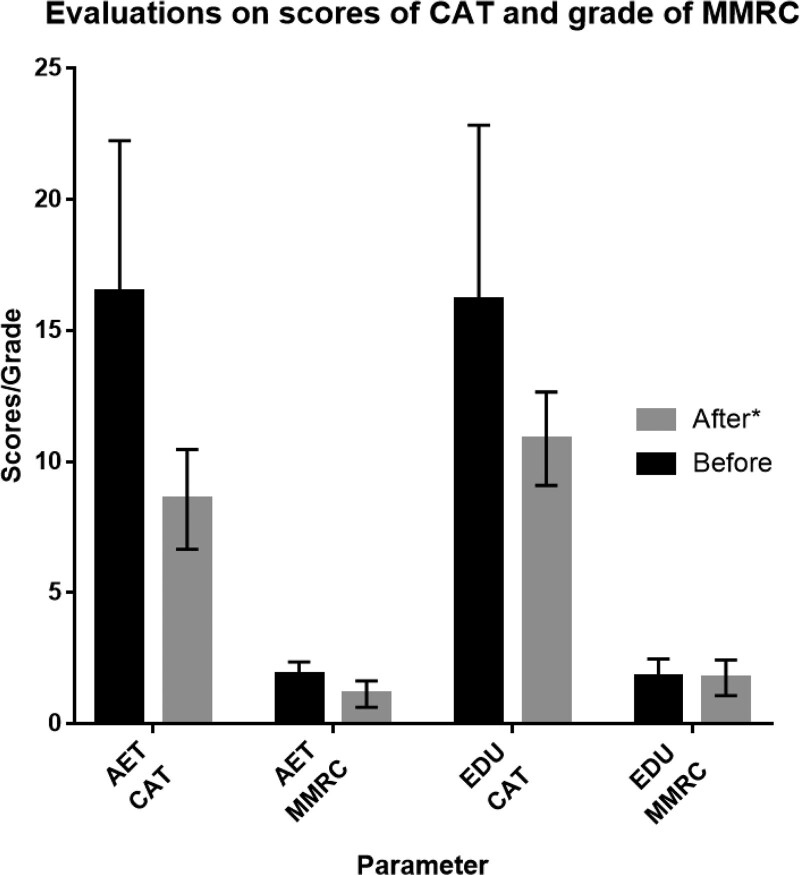
Evaluation scores of CAT and grade of mMRC. Change in CAT scores and mMRC grade in response to 6-weeks of aerobic exercise training plus education (AET) or education alone (EDU). *P < .05 for difference in change score between AET group versus EDU group. AET group = aerobic exercise training plus self-education group, CAT = chronic obstructive pulmonary disease assessment test, EDU group = self-education only group, mMRC = modified British medical research council.

The 6MWD results and the quality of life appraised by the CAT and mMRC grades were measured throughout the study and showed a distinct improvement in the AET group. In addition, a marked improvement in mMRC grade and CAT score was also observed in the AET group (*P* < .05). The improvement in the AET group was significantly higher after the 8-weeks’ aerobic exercise training compared to the EDU group (*P* < .05; Table 5; Figs. 4 and 5).

**Table 5:** Evaluations on 6MWD, scores of CAT and grade of mMRC.

Parameter	Time	EDU (n = 77)	AET (n = 79)	T-value	*P* value
6MWD	After intervention	543.50 ± 62.48	603.44 ± 77.65*	5.43	.01
CAT	After intervention	12.56 ± 2.22	8.31 ± 1.70*	−5.21	<.01
mMRC	After intervention	1.44 ± 0.51	0.94 ± 0.68*	6.49	.01

Data were analyzed with 2 independent samples *t* test and the mean values are presented.

6MWD = 6 minutes walking distance, CAT = chronic obstructive pulmonary disease assessment test, mMRC = modified British medical research council.

* *P* < .05 for difference in change score between AET group versus EDU group.

### 3.4. T lymphocyte subsets

**Figure 6.**
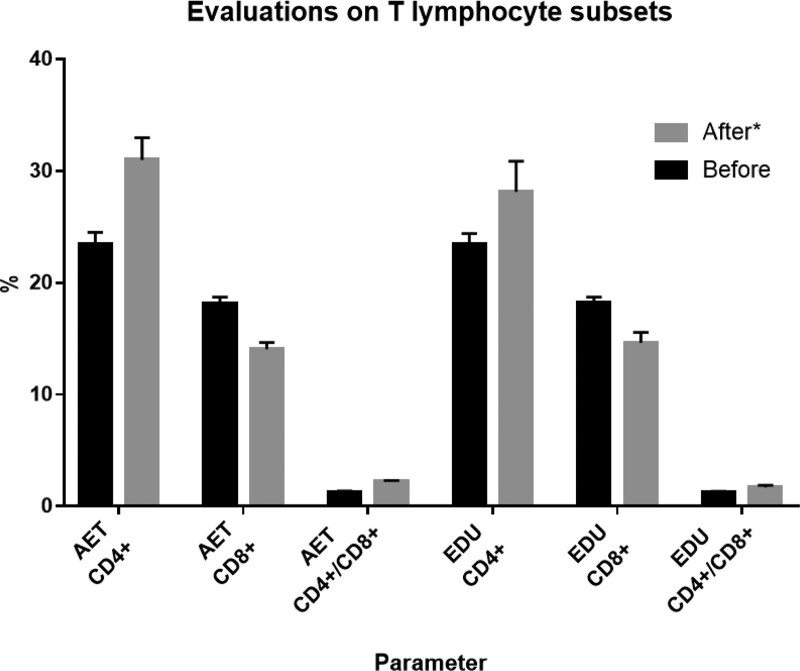
Evaluation on T lymphocyte subsets. Change in T lymphocyte subsets in response to 6-weeks of aerobic exercise training plus education (AET) or education alone (EDU). *P < .05 for difference in change score between AET group versus EDU group. AET group = aerobic exercise training plus self-education group, EDU group = self-education only group.

There was no statistically significant difference between the 2 groups of T lymphocyte subsets (CD4+, CD8+, and CD4+/CD8+) before the intervention (*P* > 0. 05). The AET group of T lymphocyte subsets changed significantly after the training compared to EDU group (*P* < .05; Table 6; Fig. 6).

**Table 6:** Evaluations on T lymphocyte subsets.

Parameter(%)	Time	EDU (n = 77)	AET (n = 79)	T-value	*P* value
CD4+	Prior to intervention	23.69 ± 1.18	23.57 ± 1.55	−0.51	.61
	After intervention	27.98 ± 2.48	30.63 ± 2.75*	6.49	.01
CD8+	Prior to intervention	18.40 ± 0.46	18.36 ± 0.42	−0.56	.67
	After intervention	17.63 ± 0.94	14.22 ± 0.76*	−25.72	<.01
CD4+/CD8+	Prior to intervention	1.29 ± 0.07	1.28 ± 0.08	−0.34	.73
	After intervention	1.59 ± 0.19	2.15 ± 0.16*	20.72	.01

All data are presented as mean ± standard deviation. Data were analyzed with 2 independent samples *t* tests.

CD4+ = cluster of differentiation 4, CD4+/CD8+ = cluster of differentiation 4 ratio cluster of differentiation 8, CD8+ = cluster of differentiation 8.

* *P* < .05 for difference in change score between AET group versus EDU group.

### 3.5. Safety variables and adverse events

Comparing the consequences of the 2 groups before and after the intervention, it had very little influence on human vital signs, such as heart rate, respiration rate, and blood pressure (data not shown), which indicates that aerobic exercise training has no adverse reactions and is safe in clinical treatment with persistent use.

## 4. Discussion

COPD is a chronic progressive disease. Its unique pathological changes are mainly seen in chronic inflammation of the airway, pulmonary parenchyma, and pulmonary vessels as well as structural changes caused by repeated injury and repair.^[17]^ The lung function will be damaged in varying degrees based on the fact that the course of COPD is longer. In addition to drug treatment, pulmonary rehabilitation with non-drug treatment is essential. The results of this study confirm that aerobic exercise training can improve lung function, reduce the expression of inflammatory factors, and relieve clinical symptoms.

Patients in the AET group showed significantly increased physical activity and decreased fatigue severity, indicating that COPD patients benefited from the aerobic exercise-training program compared to their baseline. Not only did the general disease harshness decline, but fewer AEs occurred in these patients. This finding also supports and explains the improvements in exercise training reported in previous studies.^[18–20]^ The aerobic exercise program used in the trial was a combination of resistance exercises and traditional Chinese exercises. The most significant difference in this study was in the frequency of participation. Increases in the frequency of participation were observed in previous reports, and it differs from 3 to 12 weeks.^[21]^ The results from this trial indicates that 8-weeks of aerobic exercise training also tends to be beneficial for COPD patients at a stable stage.

Moreover, coughing, sputum production, dyspnea and other symptoms showed a great decline in the AET group. We suspect that aerobic exercise training did improve personal quality and stamina exercise in daily activities, which were demonstrated to significantly enhance in 6 MWD and decrease mMRC and CAT scores. Lower mMRC and CAT questionnaire scores were obtained in the AET group. This indicates that aerobic exercise training slows down the development of COPD.

Previous information referring to the observation of effectiveness of aerobic exercise intervention in COPD patients shows a shortage of patients with a systematic evaluation. The conclusions found in earlier studies have indicated that aerobic exercise training may be safe and valid for patients with stable COPD, but methods of effectiveness only included a 6MWD and some other quality assessments.^[22–24]^ The immunological mechanism of aerobic exercise training in COPD has not reached an agreement, which may be an essential point in checking the efficacy of aerobic exercise training in patients. CD4+ and CD8+ are important in the immune and inflammatory responses.^[25]^ CD4+, as an assistant and inducer of T lymphocytes, could promote B cell-producing antibodies and strengthen the function of macrophage activation.^[26]^ CD8+ cell, as curb and killer T cells, have the opposite effect on CD4+ cells.^[27]^ Thus, the balance of CD4+/CD8+ cells in the body can ensure the normal state of the immune system and is likely to induce immune dysfunction.^[28]^ This result showed that the AET group with peripheral blood T lymphocyte subsets had significant differences compared with the EDU group, indicating that aerobic exercise training may adjust the T cell subgroup number and proportion to improve the body immune function. The outcomes of the study also agreed with those of previous studies.^[29]^

During aerobic exercise training, the body demand for energy and oxygen increases, which can increase ventricular volume, enhance myocardial contractility, and reduce oxidative stress and fiber formation.^[30,31]^ It can also enhance respiratory exercise, cause lung volume expansion, and reduce lung inflammation.^[32,33]^ The decrease in diaphragm function in patients with COPD is a critical factor for the decrease in ventilation efficiency and deterioration in quality of life.^[32]^ Aerobic exercise training, which consumes oxygen and energy, can effectively improve diaphragm function and cardiopulmonary endurance, relieve respiratory muscle fatigue, and reduce inefficient ventilation, thereby improving lung function and reducing symptoms such as fatigue and shortness of breath.^[34,35]^

Although the pathogenesis of COPD has not been completely determined, a large number of studies have shown that^[36–38]^ immune imbalance in patients with COPD is likely to mediate the expression of inflammatory factors and lead to an inflammatory response, which can lead to the aggravation and deterioration of COPD. It is worth noting that a large amount of CD4+ and CD8+ cell infiltration can be seen in the peripheral airways of lung specimens in patients with COPD, which may be the target of potential intervention therapy.^[39]^ With the purpose of slowing down the progression of COPD and reducing AE, aerobic exercise training can reduce the cytokines produced by inflammatory cells,^[40]^ increase the count of CD4+ cells and the ratio of CD4+/CD8+, improve immunity,^[41]^ as well as reducing the expression of related inflammatory factors.^[42]^ Given that benefit of aerobic exercise training, we also should come to know that, the mechanism of aerobic exercise training on immune and inflammatory factors in patients with COPD is not completely clear. Therefore, we expect animal experiments and at the molecular level for further research.

## 5. Limitations

Regarding the limitation of this retrospective study is a small sample, it prevents the conclusions from widespread generalization. In addition, the reporting biases exist during the online videos or phone calls. Further work is necessitated to work out if the conclusions would be appropriate to a big sample or to patients with other pulmonary diseases.

## 6. Conclusion

In general, this is a retrospective study demonstrating that the aerobic exercise training plays an effective and safe role in strengthening the therapies of stable COPD via decreasing the occurrence of AE, as well as the potential benefits of improving the immune system.

## Acknowledgments

We wish to thank Teacher Xue in English writing and thank Profess Lao for his advice on experimental design.

## Author contributions

**Conceptualization:** Qigang Zeng.

**Data curation:** Wentao Fang, Shuling Liu.

**Investigation:** Yong Dai.

**Project administration:** Yong Dai.

**Supervision:** Chenggong Wei.

**Validation:** Wangwang Liao, Chenxia Duan.

**Visualization:** Wentao Fang, Shuling Liu.

**Writing – original draft:** Wangwang Liao, Chenxia Duan.

**Writing – review & editing:** Qigang Zeng, Chenggong Wei.
